# *Borrelia*, *Ehrlichia*, and *Rickettsia* spp. in Ticks Removed from Persons, Texas, USA

**DOI:** 10.3201/eid1603.091333

**Published:** 2010-03

**Authors:** Phillip C. Williamson, Peggy M. Billingsley, Glenna J. Teltow, Janel P. Seals, Meredith A. Turnbough, Samuel F. Atkinson

**Affiliations:** University of North Texas Health Science Center, Fort Worth, Texas, USA (P.C. Williamson, P.M. Billingsley, J.P. Seals); Texas Department of State Health Services, Temple, Texas, USA (G.J. Teltow); University of North Texas, Denton, Texas, USA (M.A. Turnbough, S.F. Atkinson)

**Keywords:** tick-borne disease, Borrelia, Rickettsia, Ehrlichia, Candidatus Borrelia lonestari, spotted fever, ehrlichiosis, parasites, bacteria, research

## Abstract

Data regarding the type, frequency, and distribution of tick-borne pathogens and bacterial agents are not widely available for many tick species that parasitize persons in the southern United States. We therefore analyzed the frequency and identity of pathogens and bacterial agents in ticks removed from humans and subsequently submitted to the Texas Department of State Health Services, Zoonosis Control Program, from October 1, 2004, through September 30, 2008. The data showed associations of bacterial agents and potential vectors. Tick-related illnesses may pose unidentified health risks in areas such as Texas, where incidence of human disease related to tick bites is low but well above zero and where ticks are not routinely suspected as the cause of disease. Cause, treatment, and prevention strategies can be better addressed through collecting sufficient data to establish baseline assessments of risk.

## Abstract 2

Some tick-borne agents may pose yet-unknown public health risks.

Data concerning the full distribution of tick-borne agents and their potential relationship to both emerging and characterized illnesses in the southern United States are not widely available. Persons who become ill after a tick bite may be at increased risk because a tick bite may not be considered as the source of the pathogen and because of the length of time that febrile illness may elude effective treatment. Detailed knowledge of the causative agents, their distribution, and their relationship to potential vectors is also lacking. Most tick survey data for microorganisms in the genera *Borrelia*, *Rickettsia*, and *Ehrlichia* have been collected in areas where the associated diseases are considered endemic. Lyme disease, Rocky Mountain spotted fever, or human monocytotropic ehrlichiosis are not considered to be endemic to Texas. Studies of microorganisms carried in ticks in non–disease-endemic areas might provide information about potentially pathogenic organisms, their vectors, and reservoirs. These data might also provide an opportunity to examine the ecology of emerging zoonoses for which different ecologic determinants for disease transmission may be present.

In 2000, the 77th Texas Legislature Subcommittee on Administration prepared a report addressing the potentially severe nature of tick-borne disease in Texas. As of October 1, 2004, the Tick-Borne Disease Research Laboratory at the University of North Texas Health Science Center (UNTHSC) became the primary facility for testing ticks submitted to the Texas Department of State Health Services (TX DSHS).

## Methods

From October 1, 2004, through September 30, 2008, tick specimens were submitted to UNTHSC through the Zoonosis Control Program of the TX DSHS. Only ticks that had been attached to a person were submitted to UNTHSC, where they were screened for the genera *Borrelia*, *Rickettsia*, and *Ehrlichia* with genus-specific PCRs. Ticks were identified to the species level by TX DSHS entomologists before being transferred to UNTHSC (*1*–*3*). Poor condition of some specimens made identification by morphologic examination difficult. Unidentified ticks were conclusively identified by molecular methods developed at UNTHSC, which used amplification of 12S rDNA (Table 1) and sequence determination (data not shown). Additionally, the identity of any tick containing an organism not previously reported in that species was also confirmed by the same molecular methods. Of all tick specimens, 10% were screened by the same molecular identification technique to verify the accuracy of morphologic identification.

**Table 1:** Nucleotide sequence of primers used for PCR screening of tick specimens removed from humans, Texas, October 1, 2004, to September 30, 2008*

Primer name	Gene	Primer sequence (5′ → 3′)	Specificity	Screen	T_M_	Reference
Tick DNA						
85F	*12S*	TTAAGCTTTTCAGAGGAATTTGCTC	Unknown	Primary	54.0	This study
225R	*12S*	TTTWWGCTGCACCTTGACTTAA	Unknown	Primary	52.7	This study
*Borrelia* spp.						
FlaLL	*flaB*	ACATATTCAGATGCAGACAGAGGT	Genus	Primary	58.3	(*4*)
FlaRL	*flaB*	GCAATCATAGCCATTGCAGATTGT	Genus	Primary	58.9	(*4*)
FlaLS	*flaB*	AACAGCTGAAGAGCTTGGAATG	Genus	Primary	57.5	(*4*)
FlaRS	*flaB*	CTTTGATCACTTATCATTCTAATAGC	Genus	Primary	53.3	(*4*)
BL-Fla 522F	*flaB*	GGTACATATTCAGATGCAGACAGAGGG	*B. lonestari*	Primary	61.3	This study
BL-Fla 1182R	*flaB*	GCACTTGATTTGCTTGTGCAATCATAGCC	*B. lonestari*	Primary	64.0	This study
BL-Fla 662F	*flaB*	CTGAAGAGCTTGGAATGCAACCTGC	*B. lonestari*	Primary	62.8	This study
BL-Fla 860R	*flaB*	GAGCTAATCCCACCTTGAGCTGG	*B. lonestari*	Primary	61.2	This study
BL-Fla 341F	*flaB*	AGCTGATGATGCTGCTGGTATGGG	Genus	Alternate	63.2	This study
BL-Fla 730R	*flaB*	GCTTGTGCTCCAGTTAGTGATGCTGG	Genus	Alternate	64.1	This study
BL-16S 227F	*16S*	TCACACTGGAACTGAGATACGGTCC	Genus	Alternate	62.1	This study
BL-16S 920R	*16S*	GAATTAAACCACATGCTCCACCGC	Genus	Alternate	61.0	This study
BL-HSP 71F	*groEL*	CTTATGTTGAAGGAATGCAATTTGA	*B. lonestari*	Alternate	55.6	This study
BL-HSP 271R	*groEL*	CAATATCTTCAGCAATAATTAGCAAAGGT	*B. lonestari*	Alternate	58.2	This study
*Rickettsia* spp.						
Rr.190 70P	*rompA*	ATGGCGAATATTTCTCCAAAA	Genus	Primary	52.5	(*5*)
Rr.190 602N	*rompA*	AGTGCAGCATTCGCTCCCCCT	Genus	Primary	64.9	(*5*)
BG1–21	*rompB*	GGCAATTAATATCGCTGACGG	Genus	Alternate	55.6	(*6*)
BG2–20	*rompB*	GCATCTGCACTAGCACTTTC	Genus	Alternate	55.2	(*6*)
RrCS 372	*gltA*	TTTGTAGCTCTTCTCATCCTATGGC	Genus	Alternate	59.0	(*7*)
RrCS 989	*gltA*	CCCAAGTTCCTTTAATACTTCTTTGC	Genus	Alternate	57.5	(*7*)
Primer 1	*17kDa*	GCTCTTGCAACTTCTATGTT	Genus	Alternate	52.3	(*8*)
Primer 2	*17kDa*	CATTGTTCGTCAGGTTGGCG	Genus	Alternate	57.9	(*8*)
*Ehrlichia* spp.						
Ehr DSB 330F	*dsb*	GATGATGTCTGAAGATATGAAACAAAT	Genus	Primary	55.5	(*9*)
Ehr DSB 728R	*dsb*	CTGCTCGTCTATTTTACTTCTTAAAGT	Genus	Primary	56.6	(*9*)
ECC-F	*16S*	AGAACGAACGCTGGCGGCAAGCC	Genus	Alternate	68.1	(*10*)
ECB-R	*16S*	CGTATTACCGCGGCTGCTGGCA	Genus	Alternate	65.6	(*10*)
ECAN-F	*16S*	ATTTATAGCCTCTGGCTATAGGA	*E. canis*	Alternate	54.9	(*11*)
HE1-F	*16S*	CAATTGCTTATAACCTTTTGGTTATAAAT	*E. chaffeensis*	Alternate	55.6	(*12*)
EE72-F	*16S*	AATTCCTAAATAGTCTCTGACTATT	*E. ewingii*	Alternate	52.6	(*11*)
HE3-R	*16S*	TATAGGTACCGTCATTATCTTCCCTAT	Genus	Alternate	57.6	(*13*)

*T_M_, melting temperature, °C.

Ticks were bisected laterally by using aseptic technique and a sterile scalpel blade. For independent verification of results, half of each tick was stored in 100% ethanol at –80°C. For larvae and nymphs, the entire tick was used for DNA extraction. Total DNA was isolated from the second half by using an E.N.Z.A. Mollusc DNA Isolation Kit (Omega Bio-Tek, Inc., Norcross, GA, USA) according to manufacturer’s recommended protocol. Extracted DNA was subjected to PCR that used primers for the amplification of the tick’s 12S rDNA or *Borrelia* spp., *Ehrlichia* spp., and *Rickettsia* spp. genes (Table 1).

The locations of PCR setup and PCR product handling were physically separated. Reaction setup was performed in a class II type B2 biological safety cabinet that had been cleaned with 0.6% sodium hypochlorite daily and UV irradiated for 30 min before and after each use. To minimize risk for contamination, pipettor sets were dedicated to specific functions, i.e., reagent dispensing, template isolation, PCR setup, and template handling. Certified DNA/RNase-free filter barrier tips were used to prevent aerosol contamination. PCR setup was never performed in the presence of isolation materials, and reagent handling was separated both physically and temporally from templates. PCR assays were performed in duplicate with appropriate controls.

A typical, initial PCR was performed in a 25-µL reaction volume by using 5 pmol/L of each appropriate primer in conjunction with a final reaction concentration of 1× GeneAmp PCR Buffer II (Applied Biosystems, Foster City, CA, USA), 160 ng/μL bovine serum albumin, 1.0 mmol/L MgCl_2_, 200 μmol/L of each dNTP, 1.25 U Amplitaq (Applied Biosystems), and 10 µL of template. To establish the species of the tick specimen, we amplified 12S rDNA with the following cycle parameters: 95°C for 5 min; then 40 cycles each consisting of 95°C for 30 s, 45°C for 30 s, 72°C for 60 s; and a final 72°C extension for 5 min. Thermal cycling parameters for the initial PCRs of bacterial genes were 95°C for 5 min; then 40 cycles each consisting of 95°C for 60 s, 55°C for 60 s, 72°C for 30 s; and a final 72°C extension for 5 min. Nested PCR was performed by using the same reaction setup and 1.0 µL of amplified PCR mix as template. Nested PCR setup was performed in a dedicated dead air space cabinet that had been decontaminated in the same manner as the class II type B biosafety cabinet. The thermal cycling profile for the nested reactions was 95°C for 5 min; then 30 cycles each consisting of 95°C for 60 s, 55°C for 60 s, 72°C for 60 s; and a final 72°C extension for 5 min.

Verification of amplification was performed by agarose gel electrophoresis, followed by staining with 1X SYBR Green I (BioWhittaker Molecular Applications ApS, Rockland, ME, USA). Amplicons were examined with a UVP EC3 Imaging System (UVP, LLC, Upland, CA, USA) and subsequently analyzed by VisionworksLS Image Acquisition and Analysis Software (UVP, LLC). Secondary PCR systems (Table 1) were used to confirm positive results and did not contain primers that would amplify control DNA commonly used in the laboratory. Unincorporated primers were removed from samples producing amplicons before sequence determination by using ExoSAP-IT (USB Corporation, Cleveland, OH, USA).

DNA sequencing was performed for both strands of the PCR amplicons by using a BigDye Terminator Cycle Sequencing Kit, version 3.1 (Applied Biosystems). Unincorporated dye terminators were removed before electrophoresis by using Performa DTR Gel Filtration Cartridges (Edge BioSystems, Gaithersburg, MD, USA). Capillary electrophoresis was performed by using an ABI PRISM 310 Genetic Analyzer (Applied Biosystems). Final sequence analysis and editing was performed by using Sequencer 4.7 (Gene Codes Corporation, Ann Arbor, MI, USA). Using BLASTN, version 2.2.10 (www.ncbi.nlm.nih.gov/blast/Blast.cgi), we then compared edited sequence data with genetic sequences from characterized examples of *Borrelia* spp., *Rickettsia* spp., and *Ehrlichia* spp. published in GenBank.

## Results

A total of 903 ticks, representing 11 tick species, were submitted to UNTHSC from 138 of 254 Texas counties. Of these, 144 ticks contained the DNA of at least 1 of the agents in the genera *Borrelia*, *Ehrlichia*, or *Rickettsia* (Table 2). The most common tick species submitted were *Amblyomma americanum,* followed by *Dermacentor variabilis.* Spotted fever group *Rickettsia* spp. (SFGR) were the most common bacteria detected. Genetic material from SFGR was identified in *A. americanum, A. cajennense*, *D. variabilis*, *Ixodes scapularis*, *and Rhipicephalus sanguineus* ticks. Of all tick species submitted, minimum SFGR infection rates (MIRs) were highest for *A. americanum* (20.98%) and *D. variabilis* (47.37%) ticks. The most predominant SFGR sequences amplified were identical to those of *Candidatus* Rickettsia amblyommii (AY062007). Some contained a single-nucleotide difference relative to AY062007 (data not shown). SFGR amplicons produced from *Ixodes* spp. ticks were identical to those of *I. scapularis* endosymbiont isolates (EU544296, EF689740, EF689737) and shared >99% identity with *Candidatus* Rickettsia cooleyi (AF031535) (*14*) or an uncharacterized rickettsial endosymbiont previously reported for *I. scapularis* (AB002268) ticks (*15*). Amplicons with a DNA sequence identical to that of *R. parkeri* strains (U43802) (*16*), (EF102238) (*17*), and (FJ986616) were produced by 4 *D. variabilis* and 1 *Rh. sanguineus* tick samples. Amplicons identical to *R. peacockii* (CP001227) were produced by 2 *A. americanum*, 2 *D. variabilis*, and 1 *I. scapularis* tick samples. Amplicons identical to *R. rhipicephali* (U43803) and at least 99% similar to other *R. rhipicephali* strains (EU109175, EU109177, EU109178) (*18*) were produced by 1 *Rh. sanguineus* tick sample.

**Table 2:** Number and identity of ticks submitted to University of North Texas Health Science Center by the Texas Department of State Health Services Zoonosis Control Program, October 1, 2004, to September 30, 2008*

Tick	No. positive/no. tested												
	*Borrelia* spp.				*Ehrlichia* spp.				*Rickettsia* spp.				Total
	UNE	PE	E		UNE	PE	E		UNE	PE	E		
*Amblyomma americanum*													
Adult male	0/0	1/116	0/1		0/0	0/116	0/1		0/0	25/116	0/1		26/117
Adult female	0/0	1/109	0/11		0/0	2/109	0/11		0/0	23/109	4/11		30/120
Nymph	0/0	1/92	1/27		0/0	0/92	0/27		0/0	18/92	9/27		29/119
Larva	0/0	0/11	0/0		0/0	0/11	0/0		0/0	0/11	0/0		0/11
*A. cajennense*													
Adult male	0/0	0/44	0/2		0/0	1/44	0/2		0/0	0/44	0/2		1/46
Adult female	0/0	1/56	0/3		0/0	0/56	0/3		0/0	0/56	0/3		1/59
Nymph	0/0	0/52	0/3		0/0	0/52	0/3		0/0	3/52	1/3		4/55
Larva	0/0	0/12	0/0		0/0	0/12	0/0		0/0	1/12	0/0		1/12
*A. maculatum*													
Adult male	0/0	0/7	0/0		0/0	1/7	0/0		0/0	0/7	0/0		1/7
Adult female	0/0	0/1	0/1		0/0	0/1	0/1		0/0	0/1	0/1		0/2
Nymph	0/0	0/1	0/0		0/0	0/1	0/0		0/0	0/1	0/1		0/1
Larva	0/0	0/1	0/0		0/0	0/1	0/0		0/0	0/0	0/0		0/0
*Dermacentor variabilis*													
Adult male	0/1	0/71	0/1		0/1	0/71	0/1		0/1	4/71	0/0		4/73
Adult female	0/3	1/84	0/16		0/3	0/84	0/16		0/3	6/84	1/16		8/103
Nymph	0/0	0/0	0/2		0/0	0/1	0/2		0/0	0/0	0/2		0/2
Larva	0/0	0/0	0/0		0/0	0/1	0/0		0/0	0/0	0/0		0/0
*Ixodes scapularis*													
Adult male	0/0	0/4	0/0		0/0	0/4	0/0		0/0	0/4	0/0		0/4
Adult female	0/0	0/41	0/22		0/0	0/41	0/22		0/0	26/41	6/22		32/63
Nymph	0/0	1/8	0/1		0/0	0/8	0/1		0/0	4/8	0/1		5/9
Larva	0/0		0/0		0/0	0/0	0/0		0/0	0/0	0/0		0/0
*Rhipicephalus sanguineus*													
Adult male	0/0	0/23	0/0		0/0	0/23	0/0		0/0	0/23	0/0		0/23
Adult female	0/2	0/35	0/6		0/2	0/35	0/6		0/2	1/35	0/6		1/43
Nymph	0/0	0/5	0/15		0/0	0/5	0/15		0/0	0/5	1/15		1/20
Larva	0/0	0/0	0/1		0/0		0/1		0/0		0/1		0/1
Total	0/6	6/772	1/112		0/6	4/772	0/112		0/6	111/772	22/112		144/890

*Testing by PCR. Only tick species that showed evidence of containing *Borrelia*, *Ehrlichia*, or *Rickettsia* spp. are shown. Seven specimens of *Otobius megnini,* 2 of *Amblyomma inornatum* and *Dermacentor albipictus,* and 1 each of *Dermacentor andersonii* and *Dermacentor nigrolineatus* ticks were submitted during the project period. After clarification with the submitter of the *D. andersonii* specimen, it was concluded that the tick attachment may have occurred in Colorado. UNE, unengorged; PE, partially engorged; E, engorged.

DNA sequences consistent with those of *Borrelia* spp. were derived from *A. americanum, A. cajennense*, *D. variabilis*, and *I. scapularis* ticks. The most commonly encountered *Borrelia* genetic material demonstrated at least 99% sequence identity or was identical to that of previously sequenced *Candidatus* Borrelia lonestari isolates (AY850063, AF538852) (*19*). Additionally, a borreliae *flaB* sequence was generated from 1 *D. variabilis* tick, which had 94% sequence similarity with that of *Candidatus* Borrelia texasensis (AF264901) (*20*) and sequences amplified from an uncultured *Borrelia* sp. from the bat tick *Carios kelleyi* (EF688577, EF688579) (*21*) and (EU492387). The *flaB* sequence contained 11 single-nucleotide polymorphisms relative to the corresponding section of AF264901. The *Borrelia* sp. 16S rDNA sequence generated from the same *D. variabilis* tick was also identical to that published for *Candidatus* B. texasensis (AF467976) (*20*,*22*). This tick was submitted from Webb County, the same Texas county from which the borreliae that produced GenBank sequence AF264901 were isolated. A single *I. scapularis* specimen produced the *flaB* sequence, which had 99% identity with *B. burgdorferi* (AE000783) (*23*).

Genetic data consistent with those from *Ehrlichia* spp. were observed for *A. americanum*, *A. cajennense­,* and *A. maculatum* ticks. Amplicons produced from *A. americanum* and *A. maculatum* ticks were 99% similar to the homologous region of the *E. chaffeensis* disulfide oxidoreductase gene (*dsb*) sequences in GenBank (CP000236) (*24*). A single sample from *A. cajennense* ticks produced a DNA sequence that was 97% similar to that of the CP000236 sequence and contained 8 single-nucleotide polymorphisms relative to the similar sequence. Several single-nucleotide polymorphisms locations are at the same position as nucleotide differences identified between the *dsb* gene of *E. ewingii* (AY428950) (*25*) and *E. canis* (AF403710) (*26*). The nucleotide polymorphisms found within the *dsb* gene did not change the predicted amino acid sequence in relation to *E. chaffeensis* (data not shown).

## Discussion

By screening a diverse group of Texas tick species for a range of microorganisms and potential pathogens, we identified several novel associations: *Candidatus* B. lonestari in *A. cajennense* ticks, *E. chaffeensis* in *A. cajennense* ticks, and *A. maculatum*, and *R. parkeri* in *D. variabilis* ticks (Table 3). Because the geographic distribution of diseases caused by the agents is generally characterized by the distribution of the tick vectors, these findings provide insights regarding the distributions and endemicity of several potential emerging tick-borne agents.

**Table 3:** No. ticks containing bacterial DNA sequences, Texas, October 1, 2004, to September 30, 2008*

Tick species	Bacterial agent								
	*Borrelia* spp.	*B. burgdorferi*	*Candidatus* Borrelia lonestari	*Ehrlichia chaffeensis*	*Candidatus* Rickettsia amblyommii	*Candidatus* Rickettsia cooleyi	*Rickettsia parkeri*	*R. peacockii*	*R. rhipicephali*
*Amblyomma americanum*			4	2	77			2	
*A. cajennense*			1	1	5				
*A. maculatum*				1					
*Dermacentor variabilis*	1				4		4	2	1
*Ixodes scapularis*		1				35		1	
*Rhipicephalus sanguineus*					1		1		
Total	1	1	5	4	87	35	5	5	1

*Ticks submitted to the Texas Department of State Health Services and identified by the University of North Texas Health Science Center, Tick-Borne Disease Research Laboratory. Only those tick species that showed evidence of containing *Borrelia*, *Ehrlichia*, or *Rickettsia* spp. are shown.

SFGR were the most commonly observed agents in this survey. Both *Candidatus* R. amblyommii and *Candidatus* R. cooleyi are not well studied and are of undetermined pathogenicity. Current average SFGR seropositivity in Texas residents is also unknown, yet prior estimates indicate that it is higher than would be assumed from cases of Rocky Mountain spotted fever reported to the TX DSHS (*27*). Transmission through blood products has been noted previously (*28**,**29*). Unreported subclinical infections might cause concern about local blood products and could potentially compromise immunodeficient transfusion recipients. Additionally, detection of *R. amblyommii* in questing *A. americanum* larvae suggests transovarial transmission of the microbe, and the likelihood of pathogen transmission by larvae could be magnified by their habit of mass attack (huge numbers on a single host).

An overall *Borrelia* spp. MIR of 1.1% was observed for the entire 4-year collection. Prevalence of *Candidatus* B. lonestari in ticks from Texas was low. However, *Candidatus* B. lonestari sequences were detected in *A. americanum* ticks regardless of geographic origin. The MIR was slightly higher for *A. americanum* (2.53%) ticks during periods when that tick was the most abundant species parasitizing humans (October 1, 2007 through October 1, 2008). These rates are within ranges previously established in the literature (*30*–*32*). A single isolate of *Candidatus* B. lonestari was observed in *A. cajennense* ticks. This represents the potential for *Candidatus* B. lonestari to use hard ticks of species other than *A. americanum* in its maintenance cycle and suggests that *Candidatus* B. lonestari may occur in areas outside the natural distribution of *A. americanum* ticks. An MIR of 1.3% for *Borrelia* spp. was found for in *D. variabilis* and may indicate the presence of uncharacterized borreliae strains in Texas tick populations.

*A. cajennense* ticks have been associated with *E. ruminantium* (*33*) and spotted fever group *Rickettsia* spp. (*34*). According to seropositivity in a human population in Argentina, these ticks have also been suspected of transmitting ehrlichiosis (*35*). The presence of *E. chaffeensis* in an *A. cajennense* tick seems novel. Long et al. (*13*) suggest an *E. ewingii* MIR of 7.6% in southcentral Texas *A. americanum* ticks. Similar results for *Ehrlichia* spp. in *A. cajennense* tick populations may be plausible.

Screening ticks for a range of bacterial agents has provided several additional associations. These findings provide insights regarding the distributions and endemicity of potentially pathogenic and emerging tick-borne agents. Some of these tick-borne agents may pose an unknown health risk. Because of the wide distribution of these ticks, accurate assessments of the frequency of bacterial agents in these tick populations, their potential for causing human disease, and the ability for these tick species to act as competent vectors are warranted. Continued study and monitoring will play a vital role in public health assessment for related disease risks.
